# Crystal structure of the magnesium salt of the herbicide 2,4-D: penta­aqua­[(2,4-di­chloro­phenoxy)acetato-κ*O*]magnesium (2,4-di­chloro­phen­oxy)acetate hemihydrate

**DOI:** 10.1107/S1600536814019357

**Published:** 2014-09-03

**Authors:** Graham Smith

**Affiliations:** a Science and Engineering Faculty, Queensland University of Technology, GPO Box 2434, Brisbane, Queensland 4001, Australia

**Keywords:** crystal structure, magnesium complex, phen­oxy herbicide, (2,4-di­chloro­phen­oxy)acetic acid, hydrogen bonding

## Abstract

In the crystal structure of the title magnesium salt of the phen­oxy herbicide (2,4-di­chloro­phen­oxy)acetic acid (2,4-D), [Mg(C_8_H_5_Cl_2_O_3_)(H_2_O)_5_](C_8_H_5_Cl_2_O_3_)·0.5H_2_O, the discrete cationic MgO_6_ complex unit comprises a carboxyl­ate O-donor from a monodentate 2,4-D anionic ligand and five water mol­ecules, resulting in a slightly distorted octa­hedral coordination sphere. The free 2,4-D anions are linked to the complex units through duplex water–carboxyl­ate O—H⋯O hydrogen bonds through the coordinating water mol­ecules. In the crystal, inter-unit O—H⋯O hydrogen-bonding inter­actions involving coordinating water mol­ecules as well as the solvent water mol­ecule (occupancy 0.5) with carboxyl­ate O-atom acceptors, give a layered structure lying parallel to (001), in which π–π ligand–cation inter­actions [minimum ring centroid separation = 3.6405 (17) Å] and a short O—H⋯Cl inter­action are also found.

## Abstract 2

In the hydrogen-bonded structure of the Mg salt of the herbicide (2,4-di­chloro­phen­oxy)acetic acid (2,4-D), the metal atom is octa­hedrally coordinated by a monodentate 2,4-D ligand and five water mol­ecules, with a 2,4-D^−^ counter-anion and half a solvent water mol­ecule completing the crystal structure.

## Chemical context   

The phen­oxy­acetic acids comprise an important group of chemicals which has among its members those ring-substituted representatives having selective herbicidal activity, *e.g.* the commercial but in some cases, now prohibited (2,4-di­chloro­phen­oxy)acetic acid (2,4-D), (2,4,5-tri­chloro­phen­oxy)acetic acid (2,4,5-T) and (4-chloro-2-methyl­phen­oxy)acetic acid (MCPA) (O’Neil, 2002 ▶; Zumdahl, 2010 ▶; Cobb & Reade, 2011 ▶). Of inter­est have also been the structures of the metal complexes with these acids, including those with magnesium in which the monoanionic phen­oxy­acetate ligands (*L*) display a variety of coordination modes, all based on an octa­hedral MgO_6_ metal stereochemistry. These include discrete monomeric {[Mg*L*_2_(H_2_O)_4_] [*L* = 2-(2-fluoro­phen­oxy)acetate (Kennard *et al.*, 1986 ▶) and *L* = MCPA^−^ (Smith *et al.*, 1981 ▶)] and [Mg*L*(H_2_O)_5_]·*L* [*L* = 2,4,5-T^−^ (Smith *et al.*, 1982 ▶)]} or polymeric {[Mg*L*_2_(H_2_O)_2_]}_*n*_ [*L* = phen­oxy­acetate, (4-chloro­phen­oxy)acetate or (4-fluoro­phen­oxy)acetate] (Smith *et al.*, 1980 ▶; Smith, 2012 ▶)}. The title complex, [Mg(C_8_H_5_Cl_2_O_3_)(H_2_O)_5_](C_8_H_5_Cl_2_O_3_)·0.5H_2_O, was obtained from the reaction of 2,4-D with MgCO_3_ in aqueous ethanol and its crystal structure is reported herein.
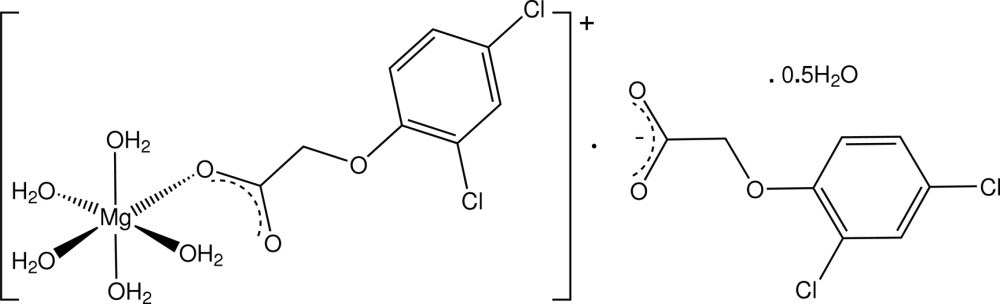


## Structural commentary   

**Figure 1:**
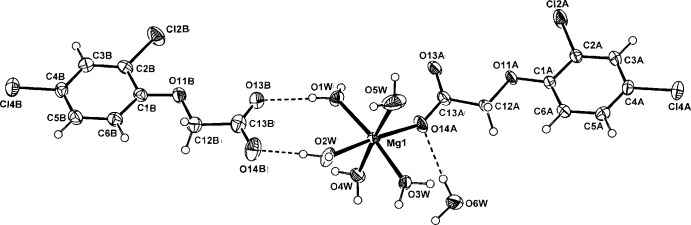
Mol­ecular configuration and atom-naming scheme for the title compound, with displacement ellipsoids drawn at the 40% probability level. Inter-species hydrogen bonds are shown as dashed lines.

In the title complex (Fig. 1 ▶), the discrete MgO_6_ complex units have, as expected, essentially octa­hedral stereochemistry [Mg—O bond length range = 2.031 (2)–2.094 (2) Å], comprising a carboxyl­ate O-donor from a monodentate 2,4-D^−^ ligand and five water mol­ecules. The free 2,4-D^−^ counter-anion is linked to the complex unit through an unusual duplex water–carboxyl­ate O—H⋯O hydrogen-bonding association involving the coordinating water mol­ecules O1*W* and O2*W* (Table 1 ▶), giving a cyclic ring motif incorporating the Mg^2+^ cation [graph set 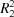
(8)]. Except for the presence of the hemihydrate mol­ecule of solvation, the title complex is very similar to that of the Mg complex with the analogous phen­oxy herbicide, (2,4,5-tri­chloro­phen­oxy)acetic acid (Smith *et al.*, 1982 ▶).

In the present complex, both 2,4-D species are essentially planar [defining torsion angles for the oxo­acetic acid side chain (C1*A/B*—O11*A/B*—C12*A/B*—C13*A/B* and O11*A/B*—C12*A/B*—C13*A/B*—O14*A/B*) being 179.0 (2) and 174.8 (2)° (ligand *A*), and 175.7 (2) and 178.7 (2)° (anion *B*), respectively]. This contrasts with the parent acid 2,4-D (Smith *et al.*, 1976 ▶), in which the oxo­acetic acid side chain adopts a synclinal conformation (benzene ring to carboxyl group dihedral angle = 75.2°).

## Supra­molecular features   

**Figure 2:**
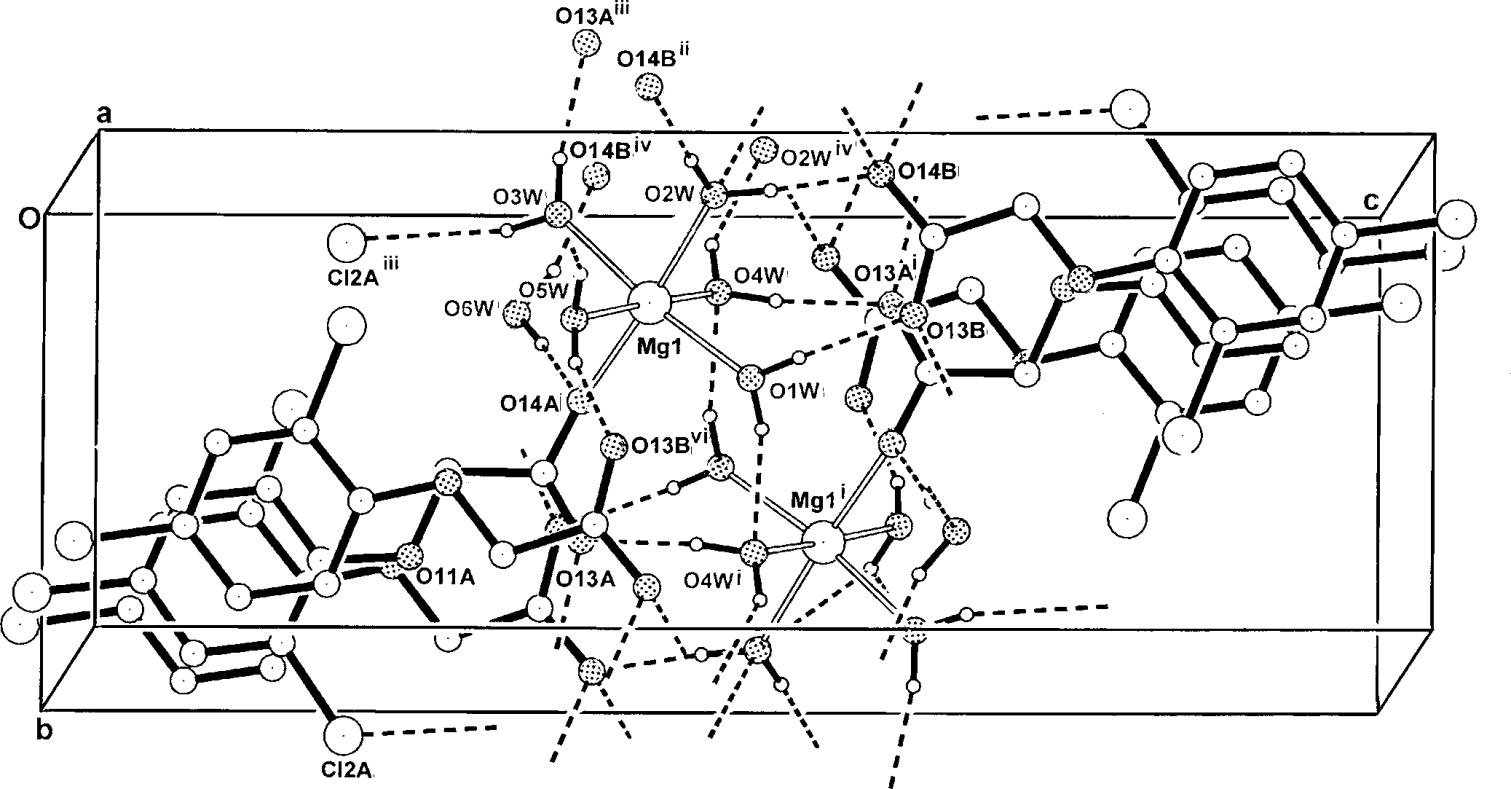
The two-dimensional hydrogen-bonded structure of the title compound in the unit cell, viewed down the *a* axis. Non-associative H atoms have been omitted. For symmetry codes, see Table 1 ▶.

In the crystal of the title compound, inter-unit O—H⋯O hydrogen-bonding inter­actions (Table 1 ▶) involving all coordinating water mol­ecules, as well as the hemihydrate solvent mol­ecule, with carboxyl­ate O-atom acceptors, give a layered structure lying parallel (001) (Fig. 2 ▶). Within these layers, weak π–π inter­actions between centrosymmetrically related 2,4-D ligand–anion species *A*⋯*B* ^i^ are also found. The 2,4-D^−^ mol­ecules lie parallel to (10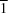) and have a minimum ring centroid separation of 3.6405 (17) Å. A short O3*W*—H⋯Cl2*A* ^iii^ inter­action [3.345 (2) Å] is also observed [for symmetry codes (i) and (iii), see: Table 1 ▶].

## Synthesis and crystallization   

The title compound was synthesized by the addition of excess MgCO_3_ to 15 ml of a hot aqueous solution of (2,4-di­chloro­phen­oxy)acetic acid (0.1 mmol) in ethanol–water (1:10 *v*/*v*). After completion of the reaction, excess MgCO_3_ was removed by filtration and the solution was allowed to evaporate at room temperature, providing colourless prisms of the title compound from which a specimen was cleaved for the X-ray analysis.

## Refinement details   

Crystal data, data collection and structure refinement details are summarized in Table 2 ▶. H atoms on all water mol­ecules were located in difference Fourier maps. Their positional parameters were refined with restraints [O—H = 0.90 (2) Å], with *U*_iso_(H) = 1.5*U*_eq_(O). Other H atoms were included in the refinement at calculated positions (aromatic C—H = 0.95 Å or methyl­ene 0.99 Å), with *U*_iso_(H) = 1.2*U*_eq_(C), using a riding-model approximation. The site-occupancy factor for the water mol­ecule of solvation was determined as 0.502 (4) and was subsequently fixed at 0.50.

## Supplementary Material

Crystal structure: contains datablock(s) global, I. DOI: 10.1107/S1600536814019357/wm5045sup1.cif

Structure factors: contains datablock(s) I. DOI: 10.1107/S1600536814019357/wm5045Isup2.hkl

CCDC reference: 1021287

Additional supporting information:  crystallographic information; 3D view; checkCIF report

## Figures and Tables

**Table 1:** Hydrogen-bond geometry (Å, °)

*D*—H⋯*A*	*D*—H	H⋯*A*	*D*⋯*A*	*D*—H⋯*A*
O1*W*—H11*W*⋯O13*B*	0.86 (3)	1.92 (3)	2.772 (3)	171 (3)
O1*W*—H12*W*⋯O4*W* ^i^	0.87 (3)	2.09 (3)	2.939 (3)	165 (3)
O2*W*—H21*W*⋯O14*B*	0.88 (2)	1.75 (2)	2.623 (3)	176 (3)
O2*W*—H22*W*⋯O14*B* ^ii^	0.87 (3)	1.88 (3)	2.754 (3)	173 (3)
O3*W*—H31*W*⋯O13*A* ^iii^	0.87 (2)	1.80 (2)	2.656 (3)	167 (3)
O3*W*—H32*W*⋯Cl2*A* ^iii^	0.87 (3)	2.50 (3)	3.345 (2)	165 (3)
O4*W*—H41*W*⋯O13*A* ^i^	0.89 (2)	1.77 (2)	2.652 (3)	172 (4)
O4*W*—H42*W*⋯O2*W* ^iv^	0.88 (2)	2.19 (3)	2.980 (3)	151 (3)
O5*W*—H51*W*⋯O6*W* ^v^	0.90 (5)	1.90 (6)	2.543 (5)	127 (4)
O5*W*—H52*W*⋯O13*B* ^vi^	0.88 (4)	1.86 (4)	2.708 (4)	162 (4)
O6*W*—H61*W*⋯O14*B* ^iv^	0.91 (6)	1.77 (6)	2.654 (5)	162 (7)
O6*W*—H62*W*⋯O14*A*	0.90 (6)	2.12 (5)	3.006 (5)	168 (5)

Symmetry codes: (i) 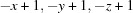; (ii) 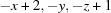; (iii) 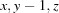; (iv) 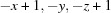; (v) 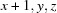; (vi) 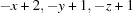.

**Table 2:** Experimental details

Crystal data	
Chemical formula	[Mg(C_8_H_5_Cl_2_O_3_)(H_2_O)_5_](C_8_H_5_Cl_2_O_3_)·0.5H_2_O
*M*_r_	563.44
Crystal system, space group	Triclinic, *P* 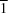
Temperature (K)	200
*a*, *b*, *c* (Å)	7.3551 (6), 7.6579 (5), 20.7878 (14)
α, β, γ (°)	91.266 (6), 94.341 (6), 94.250 (6)
*V* (Å^3^)	1163.84 (14)
*Z*	2
Radiation type	Mo *K*α
μ (mm^−1^)	0.59
Crystal size (mm)	0.40 × 0.12 × 0.10

Data collection	
Diffractometer	Oxford Diffraction Gemini-S CCD detector
Absorption correction	Multi-scan (*CrysAlis PRO*; Agilent, 2013 ▶)
*T*_min_, *T*_max_	0.970, 0.980
No. of measured, independent and observed [*I* > 2σ(*I*)] reflections	7636, 4575, 3458
*R*_int_	0.029
(sin θ/λ)_max_ (Å^−1^)	0.617

Refinement	
*R*[*F* ^2^ > 2σ(*F* ^2^)], *wR*(*F* ^2^), *S*	0.050, 0.107, 1.04
No. of reflections	4575
No. of parameters	334
No. of restraints	12
H-atom treatment	H atoms treated by a mixture of independent and constrained refinement
Δρ_max_, Δρ_min_ (e Å^−3^)	0.69, −0.51

Computer programs: *CrysAlis PRO* (Agilent, 2013 ▶), *SHELXS97* and *SHELXL97* (Sheldrick, 2008 ▶) within *WinGX* (Farrugia, 2012 ▶), and *PLATON* (Spek, 2009 ▶).

## supplementary crystallographic information

### Crystal data

**Table:**

[Mg(C_8_H_5_Cl_2_O_3_)(H_2_O)_5_](C_8_H_5_Cl_2_O_3_)·0.5H_2_O	*Z* = 2
*M**_r_* = 563.44	*F*(000) = 578
Triclinic, *P*1	*D*_x_ = 1.608 Mg m^−^^3^
Hall symbol: -P 1	Mo *K*α radiation, λ = 0.71073 Å
*a* = 7.3551 (6) Å	Cell parameters from 1889 reflections
*b* = 7.6579 (5) Å	θ = 3.5–27.2°
*c* = 20.7878 (14) Å	µ = 0.59 mm^−^^1^
α = 91.266 (6)°	*T* = 200 K
β = 94.341 (6)°	Lath, colourless
γ = 94.250 (6)°	0.40 × 0.12 × 0.10 mm
*V* = 1163.84 (14) Å^3^	

### Data collection

**Table:**

Oxford Diffraction Gemini-S CCD-detector diffractometer	4575 independent reflections
Radiation source: Enhance (Mo) X-ray source	3458 reflections with *I* > 2σ(*I*)
Graphite monochromator	*R*_int_ = 0.029
Detector resolution: 16.077 pixels mm^-1^	θ_max_ = 26.0°, θ_min_ = 3.3°
ω scans	*h* = −8→9
Absorption correction: multi-scan (*CrysAlis PRO*; Agilent, 2013)	*k* = −9→8
*T*_min_ = 0.970, *T*_max_ = 0.980	*l* = −16→25
7636 measured reflections	

### Refinement

**Table:**

Refinement on *F*^2^	Primary atom site location: structure-invariant direct methods
Least-squares matrix: full	Secondary atom site location: difference Fourier map
*R*[*F*^2^ > 2σ(*F*^2^)] = 0.050	Hydrogen site location: inferred from neighbouring sites
*wR*(*F*^2^) = 0.107	H atoms treated by a mixture of independent and constrained refinement
*S* = 1.04	*w* = 1/[σ^2^(*F*_o_^2^) + (0.0344*P*)^2^ + 0.6822*P*] where *P* = (*F*_o_^2^ + 2*F*_c_^2^)/3
4575 reflections	(Δ/σ)_max_ = 0.001
334 parameters	Δρ_max_ = 0.69 e Å^−^^3^
12 restraints	Δρ_min_ = −0.51 e Å^−^^3^

### Special details

**Table:**

Geometry. Bond distances, angles *etc*. have been calculated using the rounded fractional coordinates. All su's are estimated from the variances of the (full) variance-covariance matrix. The cell e.s.d.'s are taken into account in the estimation of distances, angles and torsion angles
Refinement. Refinement of *F*^2^ against ALL reflections. The weighted *R*-factor *wR* and goodness of fit *S* are based on *F*^2^, conventional *R*-factors *R* are based on *F*, with *F* set to zero for negative *F*^2^. The threshold expression of *F*^2^ > σ(*F*^2^) is used only for calculating *R*-factors(gt) *etc*. and is not relevant to the choice of reflections for refinement. *R*-factors based on *F*^2^ are statistically about twice as large as those based on *F*, and *R*- factors based on ALL data will be even larger.

### Fractional atomic coordinates and isotropic or equivalent isotropic displacement parameters (Å^2^)

**Table:**

	*x*	*y*	*z*	*U*_iso_*/*U*_eq_	Occ. (<1)
Cl2A	0.32241 (15)	1.10346 (11)	0.21550 (4)	0.0581 (3)	
Cl4A	0.09192 (10)	0.77979 (11)	−0.01112 (4)	0.0416 (3)	
Mg1	0.64072 (13)	0.28185 (12)	0.42864 (4)	0.0272 (3)	
O1W	0.7607 (3)	0.4533 (3)	0.50028 (11)	0.0392 (8)	
O2W	0.7909 (3)	0.0906 (3)	0.47054 (11)	0.0428 (8)	
O3W	0.5253 (3)	0.0856 (3)	0.36448 (10)	0.0384 (7)	
O4W	0.4292 (3)	0.2243 (3)	0.48859 (10)	0.0354 (7)	
O5W	0.8325 (4)	0.3474 (5)	0.36472 (14)	0.0803 (14)	
O11A	0.3737 (3)	0.7518 (2)	0.26146 (9)	0.0311 (6)	
O13A	0.5235 (3)	0.7438 (3)	0.38384 (10)	0.0394 (8)	
O14A	0.4784 (3)	0.4541 (3)	0.38457 (11)	0.0523 (9)	
C1A	0.3065 (4)	0.7496 (4)	0.19790 (13)	0.0269 (9)	
C2A	0.2754 (4)	0.9101 (4)	0.17018 (13)	0.0291 (9)	
C3A	0.2087 (4)	0.9207 (4)	0.10640 (14)	0.0314 (9)	
C4A	0.1726 (4)	0.7682 (4)	0.06967 (13)	0.0299 (9)	
C5A	0.2025 (4)	0.6081 (4)	0.09524 (14)	0.0339 (10)	
C6A	0.2687 (4)	0.5993 (4)	0.15926 (14)	0.0322 (10)	
C12A	0.3942 (4)	0.5832 (4)	0.28841 (13)	0.0308 (9)	
C13A	0.4716 (4)	0.5993 (4)	0.35773 (14)	0.0322 (10)	
Cl2B	1.16172 (14)	0.63544 (11)	0.80594 (4)	0.0560 (3)	
Cl4B	1.41057 (11)	0.24035 (12)	0.99966 (4)	0.0459 (3)	
O11B	1.1112 (3)	0.3112 (3)	0.73144 (10)	0.0377 (7)	
O13B	0.9449 (3)	0.3560 (3)	0.61359 (11)	0.0479 (8)	
O14B	0.9418 (3)	0.0723 (4)	0.58839 (11)	0.0584 (10)	
C1B	1.1820 (4)	0.2850 (4)	0.79283 (14)	0.0304 (10)	
C2B	1.2150 (4)	0.4327 (4)	0.83429 (15)	0.0324 (10)	
C3B	1.2847 (4)	0.4191 (4)	0.89740 (14)	0.0341 (10)	
C4B	1.3236 (4)	0.2574 (4)	0.91982 (14)	0.0324 (10)	
C5B	1.2948 (4)	0.1096 (4)	0.88026 (15)	0.0354 (10)	
C6B	1.2253 (4)	0.1242 (4)	0.81655 (15)	0.0356 (10)	
C12B	1.0691 (4)	0.1584 (4)	0.69131 (14)	0.0375 (11)	
C13B	0.9786 (4)	0.2045 (5)	0.62639 (15)	0.0412 (11)	
O6W	0.1369 (6)	0.2203 (6)	0.3500 (2)	0.0411 (17)	0.500
H3A	0.18810	1.03100	0.08830	0.0380*	
H5A	0.17800	0.50420	0.06930	0.0410*	
H6A	0.28850	0.48840	0.17700	0.0390*	
H11W	0.828 (4)	0.423 (5)	0.5331 (13)	0.0590*	
H12A	0.47650	0.51830	0.26280	0.0370*	
H12W	0.713 (5)	0.549 (3)	0.5108 (17)	0.0590*	
H13A	0.27360	0.51580	0.28600	0.0370*	
H21W	0.838 (5)	0.089 (5)	0.5105 (10)	0.0640*	
H22W	0.869 (4)	0.035 (5)	0.4497 (16)	0.0640*	
H31W	0.511 (5)	−0.028 (2)	0.3660 (17)	0.0580*	
H32W	0.464 (4)	0.108 (5)	0.3286 (12)	0.0580*	
H41W	0.451 (4)	0.245 (5)	0.5307 (9)	0.0530*	
H42W	0.371 (4)	0.120 (3)	0.4861 (17)	0.0530*	
H51W	0.912 (6)	0.275 (6)	0.382 (3)	0.1210*	
H52W	0.900 (6)	0.447 (4)	0.363 (2)	0.1210*	
H3B	1.30550	0.52030	0.92500	0.0410*	
H5B	1.32220	−0.00140	0.89630	0.0430*	
H6B	1.20730	0.02270	0.78900	0.0430*	
H12B	0.98600	0.07460	0.71280	0.0450*	
H13B	1.18280	0.10110	0.68470	0.0450*	
H61W	0.126 (10)	0.131 (7)	0.378 (3)	0.0620*	0.500
H62W	0.230 (7)	0.296 (7)	0.365 (3)	0.0620*	0.500

### Atomic displacement parameters (Å^2^)

**Table:**

	*U*^11^	*U*^22^	*U*^33^	*U*^12^	*U*^13^	*U*^23^
Cl2A	0.1102 (8)	0.0252 (4)	0.0356 (5)	0.0087 (5)	−0.0197 (5)	0.0018 (3)
Cl4A	0.0416 (5)	0.0557 (5)	0.0268 (4)	0.0087 (4)	−0.0071 (3)	0.0001 (4)
Mg1	0.0359 (5)	0.0197 (5)	0.0251 (5)	−0.0003 (4)	−0.0017 (4)	0.0002 (4)
O1W	0.0516 (14)	0.0262 (12)	0.0373 (13)	0.0055 (10)	−0.0121 (10)	−0.0075 (10)
O2W	0.0436 (13)	0.0400 (14)	0.0434 (14)	0.0202 (11)	−0.0164 (10)	−0.0166 (11)
O3W	0.0561 (14)	0.0208 (11)	0.0348 (13)	0.0009 (10)	−0.0169 (10)	−0.0010 (10)
O4W	0.0464 (13)	0.0290 (12)	0.0309 (12)	0.0021 (10)	0.0042 (10)	0.0036 (10)
O5W	0.082 (2)	0.095 (3)	0.0573 (19)	−0.0509 (18)	0.0218 (16)	−0.0094 (17)
O11A	0.0489 (12)	0.0209 (10)	0.0232 (10)	0.0040 (9)	−0.0023 (8)	0.0071 (8)
O13A	0.0628 (15)	0.0246 (12)	0.0295 (12)	−0.0012 (10)	−0.0020 (10)	0.0065 (9)
O14A	0.0907 (19)	0.0218 (12)	0.0419 (14)	0.0064 (12)	−0.0159 (12)	0.0106 (10)
C1A	0.0289 (15)	0.0283 (16)	0.0234 (15)	0.0018 (12)	0.0010 (11)	0.0055 (12)
C2A	0.0371 (17)	0.0261 (16)	0.0238 (15)	0.0014 (13)	0.0007 (12)	0.0020 (12)
C3A	0.0342 (16)	0.0314 (17)	0.0296 (16)	0.0079 (13)	0.0013 (12)	0.0088 (13)
C4A	0.0266 (15)	0.0413 (18)	0.0216 (15)	0.0028 (13)	−0.0005 (11)	0.0024 (13)
C5A	0.0363 (17)	0.0318 (17)	0.0324 (17)	−0.0004 (13)	−0.0006 (13)	−0.0040 (13)
C6A	0.0386 (17)	0.0265 (16)	0.0312 (17)	0.0007 (13)	0.0016 (13)	0.0066 (13)
C12A	0.0438 (18)	0.0188 (15)	0.0302 (16)	0.0040 (13)	0.0015 (13)	0.0070 (12)
C13A	0.0425 (18)	0.0249 (16)	0.0299 (17)	0.0063 (13)	0.0025 (13)	0.0065 (13)
Cl2B	0.0899 (7)	0.0302 (5)	0.0493 (5)	0.0140 (4)	0.0029 (5)	0.0068 (4)
Cl4B	0.0388 (5)	0.0653 (6)	0.0332 (4)	0.0054 (4)	−0.0018 (3)	0.0070 (4)
O11B	0.0491 (13)	0.0354 (13)	0.0278 (12)	0.0029 (10)	−0.0017 (9)	0.0037 (9)
O13B	0.0471 (14)	0.0538 (16)	0.0409 (14)	−0.0040 (12)	−0.0052 (10)	0.0117 (12)
O14B	0.0687 (17)	0.0742 (19)	0.0330 (14)	0.0209 (14)	−0.0021 (11)	−0.0153 (13)
C1B	0.0288 (16)	0.0316 (17)	0.0314 (17)	0.0016 (13)	0.0049 (12)	0.0051 (13)
C2B	0.0362 (17)	0.0245 (16)	0.0380 (18)	0.0055 (13)	0.0075 (13)	0.0051 (13)
C3B	0.0339 (17)	0.0333 (18)	0.0351 (18)	0.0024 (13)	0.0040 (13)	−0.0014 (13)
C4B	0.0259 (16)	0.0419 (19)	0.0298 (17)	0.0047 (13)	0.0018 (12)	0.0058 (14)
C5B	0.0335 (17)	0.0345 (18)	0.0389 (18)	0.0049 (14)	0.0021 (13)	0.0091 (14)
C6B	0.0411 (18)	0.0278 (17)	0.0380 (18)	0.0039 (14)	0.0017 (13)	0.0024 (14)
C12B	0.0383 (18)	0.0406 (19)	0.0339 (18)	0.0043 (14)	0.0042 (13)	0.0005 (14)
C13B	0.0361 (18)	0.060 (2)	0.0287 (18)	0.0055 (17)	0.0094 (13)	−0.0006 (17)
O6W	0.044 (3)	0.032 (3)	0.047 (3)	0.000 (2)	0.003 (2)	0.004 (2)

### Geometric parameters (Å, º)

**Table:**

Mg1—O1W	2.065 (2)	O14B—C13B	1.270 (5)
Mg1—O4W	2.094 (2)	O6W—H61W	0.91 (6)
Mg1—O5W	2.053 (3)	O6W—H62W	0.90 (6)
Mg1—O14A	2.031 (2)	C1A—C6A	1.388 (4)
Mg1—O2W	2.067 (2)	C1A—C2A	1.396 (4)
Mg1—O3W	2.076 (2)	C2A—C3A	1.385 (4)
Cl2A—C2A	1.736 (3)	C3A—C4A	1.379 (4)
Cl4A—C4A	1.745 (3)	C4A—C5A	1.373 (4)
Cl2B—C2B	1.734 (3)	C5A—C6A	1.387 (4)
Cl4B—C4B	1.744 (3)	C12A—C13A	1.508 (4)
O11A—C1A	1.375 (3)	C3A—H3A	0.9500
O11A—C12A	1.432 (3)	C5A—H5A	0.9500
O13A—C13A	1.243 (4)	C6A—H6A	0.9500
O14A—C13A	1.258 (4)	C12A—H12A	0.9900
O1W—H12W	0.87 (3)	C12A—H13A	0.9900
O1W—H11W	0.86 (3)	C1B—C6B	1.386 (4)
O2W—H22W	0.87 (3)	C1B—C2B	1.402 (4)
O2W—H21W	0.88 (2)	C2B—C3B	1.381 (4)
O3W—H31W	0.870 (16)	C3B—C4B	1.375 (4)
O3W—H32W	0.87 (3)	C4B—C5B	1.378 (4)
O4W—H42W	0.88 (2)	C5B—C6B	1.393 (4)
O4W—H41W	0.89 (2)	C12B—C13B	1.520 (4)
O5W—H51W	0.90 (5)	C3B—H3B	0.9500
O5W—H52W	0.88 (4)	C5B—H5B	0.9500
O11B—C1B	1.366 (4)	C6B—H6B	0.9500
O11B—C12B	1.423 (4)	C12B—H13B	0.9900
O13B—C13B	1.235 (4)	C12B—H12B	0.9900
			
O1W—Mg1—O2W	87.62 (10)	C4A—C5A—C6A	119.5 (3)
O1W—Mg1—O3W	173.07 (10)	C1A—C6A—C5A	121.2 (3)
O1W—Mg1—O4W	87.94 (9)	O11A—C12A—C13A	111.3 (2)
O1W—Mg1—O5W	93.97 (12)	O14A—C13A—C12A	113.2 (3)
O1W—Mg1—O14A	96.53 (10)	O13A—C13A—C12A	121.7 (3)
O2W—Mg1—O3W	86.18 (9)	O13A—C13A—O14A	125.1 (3)
O2W—Mg1—O4W	90.97 (9)	C2A—C3A—H3A	121.00
O2W—Mg1—O5W	93.47 (12)	C4A—C3A—H3A	121.00
O2W—Mg1—O14A	175.40 (10)	C6A—C5A—H5A	120.00
O3W—Mg1—O4W	89.06 (9)	C4A—C5A—H5A	120.00
O3W—Mg1—O5W	89.51 (12)	C5A—C6A—H6A	119.00
O3W—Mg1—O14A	89.56 (10)	C1A—C6A—H6A	119.00
O4W—Mg1—O5W	175.23 (12)	O11A—C12A—H12A	109.00
O4W—Mg1—O14A	87.21 (10)	O11A—C12A—H13A	109.00
O5W—Mg1—O14A	88.23 (12)	C13A—C12A—H13A	109.00
C1A—O11A—C12A	115.3 (2)	H12A—C12A—H13A	108.00
Mg1—O14A—C13A	146.3 (2)	C13A—C12A—H12A	109.00
H11W—O1W—H12W	108 (3)	O11B—C1B—C2B	117.1 (3)
Mg1—O1W—H11W	124 (3)	O11B—C1B—C6B	124.8 (3)
Mg1—O1W—H12W	122 (2)	C2B—C1B—C6B	118.0 (3)
H21W—O2W—H22W	102 (3)	Cl2B—C2B—C1B	119.0 (2)
Mg1—O2W—H21W	127 (2)	Cl2B—C2B—C3B	119.6 (2)
Mg1—O2W—H22W	123 (2)	C1B—C2B—C3B	121.4 (3)
Mg1—O3W—H31W	134 (2)	C2B—C3B—C4B	119.2 (3)
Mg1—O3W—H32W	122 (2)	Cl4B—C4B—C3B	119.2 (2)
H31W—O3W—H32W	103 (3)	Cl4B—C4B—C5B	119.8 (2)
Mg1—O4W—H41W	119 (2)	C3B—C4B—C5B	121.0 (3)
Mg1—O4W—H42W	120 (2)	C4B—C5B—C6B	119.5 (3)
H41W—O4W—H42W	104 (3)	C1B—C6B—C5B	120.8 (3)
H51W—O5W—H52W	103 (4)	O11B—C12B—C13B	110.8 (3)
Mg1—O5W—H51W	93 (3)	O13B—C13B—C12B	122.0 (3)
Mg1—O5W—H52W	128 (3)	O14B—C13B—C12B	113.0 (3)
C1B—O11B—C12B	116.2 (2)	O13B—C13B—O14B	125.0 (3)
H61W—O6W—H62W	109 (6)	C2B—C3B—H3B	120.00
C2A—C1A—C6A	117.7 (3)	C4B—C3B—H3B	120.00
O11A—C1A—C6A	124.6 (3)	C4B—C5B—H5B	120.00
O11A—C1A—C2A	117.6 (2)	C6B—C5B—H5B	120.00
Cl2A—C2A—C1A	120.1 (2)	C5B—C6B—H6B	120.00
C1A—C2A—C3A	121.7 (3)	C1B—C6B—H6B	120.00
Cl2A—C2A—C3A	118.2 (2)	O11B—C12B—H12B	110.00
C2A—C3A—C4A	118.8 (3)	O11B—C12B—H13B	109.00
C3A—C4A—C5A	121.1 (3)	C13B—C12B—H13B	110.00
Cl4A—C4A—C3A	119.3 (2)	H12B—C12B—H13B	108.00
Cl4A—C4A—C5A	119.6 (2)	C13B—C12B—H12B	109.00
			
O1W—Mg1—O14A—C13A	56.7 (4)	C2A—C3A—C4A—C5A	−0.3 (4)
O3W—Mg1—O14A—C13A	−126.6 (4)	C3A—C4A—C5A—C6A	0.6 (5)
O4W—Mg1—O14A—C13A	144.3 (4)	Cl4A—C4A—C5A—C6A	179.2 (2)
O5W—Mg1—O14A—C13A	−37.1 (4)	C4A—C5A—C6A—C1A	−0.4 (4)
C12A—O11A—C1A—C2A	176.9 (3)	O11A—C12A—C13A—O13A	−5.8 (4)
C12A—O11A—C1A—C6A	−3.6 (4)	O11A—C12A—C13A—O14A	174.8 (2)
C1A—O11A—C12A—C13A	179.0 (2)	O11B—C1B—C2B—Cl2B	0.9 (4)
Mg1—O14A—C13A—O13A	−60.7 (5)	O11B—C1B—C2B—C3B	179.4 (3)
Mg1—O14A—C13A—C12A	118.7 (3)	C6B—C1B—C2B—Cl2B	−179.9 (2)
C12B—O11B—C1B—C2B	−176.6 (3)	C6B—C1B—C2B—C3B	−1.4 (4)
C12B—O11B—C1B—C6B	4.3 (4)	O11B—C1B—C6B—C5B	−179.1 (3)
C1B—O11B—C12B—C13B	175.7 (2)	C2B—C1B—C6B—C5B	1.7 (4)
C6A—C1A—C2A—C3A	0.2 (4)	Cl2B—C2B—C3B—C4B	178.9 (2)
O11A—C1A—C6A—C5A	−179.4 (3)	C1B—C2B—C3B—C4B	0.4 (5)
C2A—C1A—C6A—C5A	0.1 (4)	C2B—C3B—C4B—Cl4B	−179.7 (2)
O11A—C1A—C2A—C3A	179.7 (3)	C2B—C3B—C4B—C5B	0.3 (4)
C6A—C1A—C2A—Cl2A	−179.2 (2)	Cl4B—C4B—C5B—C6B	180.0 (2)
O11A—C1A—C2A—Cl2A	0.3 (4)	C3B—C4B—C5B—C6B	0.0 (5)
Cl2A—C2A—C3A—C4A	179.3 (2)	C4B—C5B—C6B—C1B	−1.1 (5)
C1A—C2A—C3A—C4A	−0.1 (5)	O11B—C12B—C13B—O13B	−1.9 (4)
C2A—C3A—C4A—Cl4A	−179.0 (2)	O11B—C12B—C13B—O14B	178.7 (2)

### Hydrogen-bond geometry (Å, º)

**Table:**

*D*—H···*A*	*D*—H	H···*A*	*D*···*A*	*D*—H···*A*
O1*W*—H11*W*···O13*B*	0.86 (3)	1.92 (3)	2.772 (3)	171 (3)
O1*W*—H12*W*···O4*W*^i^	0.87 (3)	2.09 (3)	2.939 (3)	165 (3)
O2*W*—H21*W*···O14*B*	0.88 (2)	1.75 (2)	2.623 (3)	176 (3)
O2*W*—H22*W*···O14*B*^ii^	0.87 (3)	1.88 (3)	2.754 (3)	173 (3)
O3*W*—H31*W*···O13*A*^iii^	0.87 (2)	1.80 (2)	2.656 (3)	167 (3)
O3*W*—H32*W*···Cl2*A*^iii^	0.87 (3)	2.50 (3)	3.345 (2)	165 (3)
O4*W*—H41*W*···O13*A*^i^	0.89 (2)	1.77 (2)	2.652 (3)	172 (4)
O4*W*—H42*W*···O2*W*^iv^	0.88 (2)	2.19 (3)	2.980 (3)	151 (3)
O5*W*—H51*W*···O6*W*^v^	0.90 (5)	1.90 (6)	2.543 (5)	127 (4)
O5*W*—H52*W*···O13*B*^vi^	0.88 (4)	1.86 (4)	2.708 (4)	162 (4)
O6*W*—H61*W*···O14*B*^iv^	0.91 (6)	1.77 (6)	2.654 (5)	162 (7)
O6*W*—H62*W*···O14*A*	0.90 (6)	2.12 (5)	3.006 (5)	168 (5)

Symmetry codes: (i) −*x*+1, −*y*+1, −*z*+1; (ii) −*x*+2, −*y*, −*z*+1; (iii) *x*, *y*−1, *z*; (iv) −*x*+1, −*y*, −*z*+1; (v) *x*+1, *y*, *z*; (vi) −*x*+2, −*y*+1, −*z*+1.
